# Cortical nitric oxide required for presynaptic long-term potentiation in the insular cortex

**DOI:** 10.1098/rstb.2023.0475

**Published:** 2024-07-29

**Authors:** Kiyofumi Yamamoto, Qi-Yu Chen, Zhaoxiang Zhou, Masayuki Kobayashi, Min Zhuo

**Affiliations:** ^1^ Department of Pharmacology, Nihon University School of Dentistry, 1-8-13 Kanda-Surugadai, Chiyoda-ku, Tokyo 101-8310, Japan; ^2^ Department of Physiology, Faculty of Medicine, University of Toronto, Medical Science Building, 1 King's College Circle, Toronto, Ontario M5S 1A8, Canada; ^3^ Zhuomin Institute for Brain Research, Qingdao 266000, People's Republic of China; ^4^ CAS Key Laboratory of Brain Connectome and Manipulation, Interdisciplinary Center for Brain Information, The Brain Cognition and Brain Disease Institute, Shenzhen Institute of Advanced Technology, Chinese Academy of Sciences Shenzhen Institute of Advanced Technology, Shenzhen 518055, People's Republic of China; ^5^ Department of Neurology, The First Affiliated Hospital of Guangzhou Medical University, Guangzhou 510130, People's Republic of China

**Keywords:** insular cortex, presynaptic long-term potentiation, nitric oxide, kainate receptor

## Abstract

Nitric oxide (NO) is a key diffusible messenger in the mammalian brain. It has been proposed that NO may diffuse retrogradely into presynaptic terminals, contributing to the induction of hippocampal long-term potentiation (LTP). Here, we present novel evidence that NO is required for kainate receptor (KAR)-dependent presynaptic form of LTP (pre-LTP) in the adult insular cortex (IC). In the IC, we found that inhibition of NO synthase erased the maintenance of pre-LTP, while the induction of pre-LTP required the activation of KAR. Furthermore, NO is essential for pre-LTP induced between two pyramidal cells in the IC using the double patch-clamp recording. These results suggest that NO is required for homosynaptic pre-LTP in the IC. Our results present strong evidence for the critical roles of NO in pre-LTP in the IC.

This article is part of a discussion meeting issue ‘Long-term potentiation: 50 years on’.

## 1. Introduction

Synaptic long-term potentiation (LTP) has been widely studied in the hippocampus, amygdala, cerebellum and many cerebral cortical areas, and it is considered to be the basic cellular model for learning and memory [1–4]. Different forms of LTP have been reported in the hippocampus and cortex regarding basic mechanisms for the induction and expression of LTP [5]. Two major forms of LTP have been reported in adult cortical synapses: *N*-methyl-d-aspartate receptor (NMDAR)-mediated postsynaptic LTP (post-LTP) and kainate receptor (KAR)-mediated presynaptic LTP (pre-LTP) [2,5,6]. Similar forms of postsynaptic LTP induced by postsynaptic NMDAR have been reported in the hippocampus. In addition, there are reports of retrograde messengers, such as nitric oxide (NO), in the CA1 region of the hippocampus [7–10]. In this case, the retrograde messengers generated by the activation of postsynaptic NMDARs are proposed to diffuse to presynaptic terminals to enhance the release of glutamate during LTP.

Among several candidates for retrograde messengers, NO receives the most attention [11]. In the CA1 region of the hippocampus, it has been proposed that NO may be produced at postsynaptic sites and then diffuses to presynaptic terminals in which it activates the cyclic guanosine monophosphate-dependent signalling pathway [7–10]. However, it has been reported that certain forms of CA1 LTP may be purely expressed by postsynaptic mechanisms, such as the modification of α-amino-3-hydroxy- 5-methyl-isoxozole propionic acid receptor (AMPAR) or the insertion of newly synthesized AMPARs [12,13]. Furthermore, a new form of KAR-dependent LTP in the hippocampus has been reported, which is purely expressed by the presynaptic mechanism [6]. There is no report of the requirement of NO in this form of pre-LTP in the hippocampus.

The insular cortex (IC) is one of the critical regions for pain perception, taste aversion and emotional disorders [14–17]. Immunohistochemical and autoradiographic results revealed that the IC, particularly the area surrounding the rhinal fissure, shows a markedly higher-level activity of NO synthase (NOS) and its associating coenzyme, nicotinamide adenine dinucleotide phosphate, in comparison with other cortical regions [18]. However, little is known concerning the specific functions of NO in the IC. Here, we show that the novel presynaptic KAR-NO pathway contributes to pre-LTP in the IC.

## 2. Methods

### (a) Animals

Adult male C57BL/6 mice (8–12 weeks old) were used in most of the experiments. Mice were housed under a 12 L : 12 D cycle (onset at 7:00 a.m.) with food and water provided ad libitum. All mouse protocols were approved by the Animal Care and Use Committee of the University of Toronto (protocol ID, 20012315 and 20012148) and the Institutional Animal Care and Use Committee at Nihon University (ID, AP19DEN028-4).

### (b) Slice preparation

The data reported below consist of results from 149 mice. We began preparing slices from a mouse that was kept in a bright room for approximately 2–3 h (9.00–10.00). Briefly, mice were deeply anaesthetized with isoflurane (5%). After decapitation, tissue blocks, including IC, were rapidly removed and stored for 2 min in ice-cold artificial cerebrospinal fluid (ACSF) containing the following (in mM): 124 NaCl, 2.5 KCl, 2 MgSO_4_, 1 NaH_2_PO_4_, 25 NaHCO_3_, 2 CaCl_2_ and 10 d-glucose. Coronal slices were cut at a thickness of 300 μm using a microslicer (VT1200S, Leica, Wetzlar, Germany). Slices were incubated at room temperature for 1 h in a submersion-type holding chamber. ACSF was continuously aerated with a mixture of 95% O_2_/5% CO_2_.

### (c) Whole-cell patch-clamp recording

The slices were placed in a recording chamber that was perfused continuously with normal ACSF at a rate of 0.8–1.0 ml min^-1^. Whole-cell patch-clamp recordings were obtained from pyramidal cells in layer II/III using visualized observation with Nomarski optics (×40, Olympus BX51W1, Tokyo, Japan) and an infrared-sensitive video camera (OYL-150, Olympus). To obtain evoked excitatory postsynaptic current (eEPSC) responses, we delivered stimulations by a bipolar tungsten stimulating electrode (50 MΩ, WE3ST10.5F3; MicroProbes, Gaithersburg, MD, USA) placed in layer V of the IC. eEPSCs were induced by repetitive stimulations at 0.02 Hz to measure the baseline in the pre-LTP experiments. In our previous study in the IC of the rat, a laser scanning photo-stimulation technique revealed that pyramidal cells located in layers II/III are not likely to receive strong excitatory inputs from a region with a perpendicular angle to cortical face but rather two different regions, dorsal and ventral sites, with the oblique angles of layer V [19,20]. Therefore, we placed the electrodes on the dorsal and ventral sites. To obtain unitary EPSC (uEPSC) responses, we carried out double whole-cell patch-clamp recording from adjacent pyramidal cells placed in layers II/III in the IC. Before uEPSC recordings, the voltage responses of pre- and post-synaptic cells were recorded by the injection of depolarizing current pulses (300 ms) to examine basic membrane properties, including repetitive firing patterns and frequency. Except for part of the experiments, presynaptic cells were recorded under current-clamp conditions during uEPSC recording to provide transmitter release from presynaptic cells. Short depolarizing current step pulses (1 ms, 2.5–4 nA) were applied to the presynaptic cells to induce action potentials. In the protocol for post-LTP and double patch-clamp recordings, eEPSCs and uEPSCs were delivered by repetitive stimulation at 0.033 Hz during the control and the period after the stimulus protocol. Induction of post-LTP and pre-LTP with double-patch recordings was performed within 10–15 min after establishing the whole-cell configuration to avoid the washout of intracellular contents that are critical for the establishment of synaptic plasticity. For the induction of pre-LTP of eEPSC, 240 paired presynaptic stimuli (with 50 ms inter-pulse intervals) were delivered at 2 Hz to the presynaptic fibres at a holding potential of −75 mV. To induce pre- and post-LTP in the IC, we used each protocol that was previously reported [6,19]. For the induction of pre-LTP of uEPSC, 240 paired current injections to induce action potentials (2.5–4 nA, with 50 ms inter-pulse intervals) were delivered at 2 Hz to the presynaptic cells. For induction of post-LTP, 80 single stimuli were delivered at 2 Hz to the presynaptic fibres at a holding potential of 30 mV. The composition of the pipette solution was as follows (in mM): 145 K-gluconate, 5 mM NaCl, 1 mM MgCl_2_, 0.2 Ethylene glycol-bis(2-aminoethylether)-N,N,N′,N′-tetraacetic acid (EGTA), 10 4-(2-Hydroxyethyl)-1-piperazine ethanesulfonic acid (HEPES), 2 Mg-Adenosine triphosphate (ATP) and 0.1 Na_3_-Guanosine-5'-triphosphate (GPT). The pipette solution had a pH of 7.3 and an osmolarity of 300 mOsm. Electrical signals were recorded with amplifiers (Multiclamp 700B, Molecular Devices, Sunnyvale, CA) and a digitizer (Digidata 1440A, Molecular Devices), observed online, and stored on a computer hard disk using Clampex (pClamp 10, Molecular Devices). For miniature EPSC (mEPSC) recordings, 50 µM D-AP5, 1 µM tetrodotoxin (TTX) and 100 µM picrotoxin were added to the perfusion solution. mEPSCs were detected at a threshold of three times the standard deviation (s.d.) of the baseline noise amplitude using event detection software. Access resistance was 15–30 MΩ and was monitored throughout the experiment. Data were discarded if access resistance changed >15% during an experiment. Data were filtered at 2 kHz and digitized at 10 kHz.

### (d) Drugs

Nomega-nitro-l-arginine methyl ester hydrochloride (L-NAME, 100–200 µM) and TTX (1 µM) were purchased from HelloBio (Princeton, NJ, USA) and added to the perfusate. Stock solution of (*S*)-1-(2-amino-2-carboxyethyl)-3-(2 -carboxy-thiophene-3-yl-methyl)-5-methylpyrimidine-2,4-dione (UBP310, 10 µM; HelloBio) and 1,4-dihydro-2,6-dimethyl-4-(2-nitrophenyl)-3,5-pyridinedicarboxylic acid dimethyl ester (nifedipine, 20 µM; Sigma-Aldrich, St Louis, MO, USA) were prepared in dimethyl sulfoxide at 10 and 20 mM, respectively. l-arginine (L-Arg; 1 mM) was purchased from Sigma-Aldrich. eEPSC amplitude during drug application was recorded and monitored online. Spermine and PAPANONOate (100 µM) were purchased from Santa Cruz Biotechnology (Dallas, TX, USA) and Abcam (Trumpington, Cambridge, UK). To obtain the steady eEPSC amplitude, we applied the drugs for 7–12 min. Other compounds were purchased from Sigma-Aldrich or Bioshop (Burlington, Ontario, Canada).

### (e) Experimental design and analysis

Clampfit (pClamp 9, Molecular Devices) was used for the analysis of electrophysiological data. Averaged amplitude and paired-pulse ratio (PPR) determined by the ratio of the peak amplitude of the second eEPSCs to that of the first eEPSCs were obtained from 8 to 12 consecutive sweeps. The events in the mEPSC recordings were detected by WDETECTA, which was kindly provided by Professor John Huguenard (Stanford University); the number of events and their amplitudes were quantitatively analysed. The synaptic responses in the mEPSC and uEPSC recordings were detected at a threshold of three times s.d. of the baseline noise amplitude using event detection software. The failure of uEPSCs was defined to be less than three times the s.d. of the baseline. The values are expressed as the mean ± standard error of the mean (s.e.m.). The error bars in bar graphs represent s.e.m. Differences in the mean values between the two groups were compared with a paired *t*‐test, Student’s *t*‐test. A Mann–Whitney *U*-test was employed for analysis of failure rate in uEPSCs. Differences with a probability (*p*) <0.05 were considered significant.

## 3. Results

### (a) Nitric oxide synthase inhibitor-sensitive long-term potentiation of excitatory synaptic responses in the insular cortex

**Figure 1.**
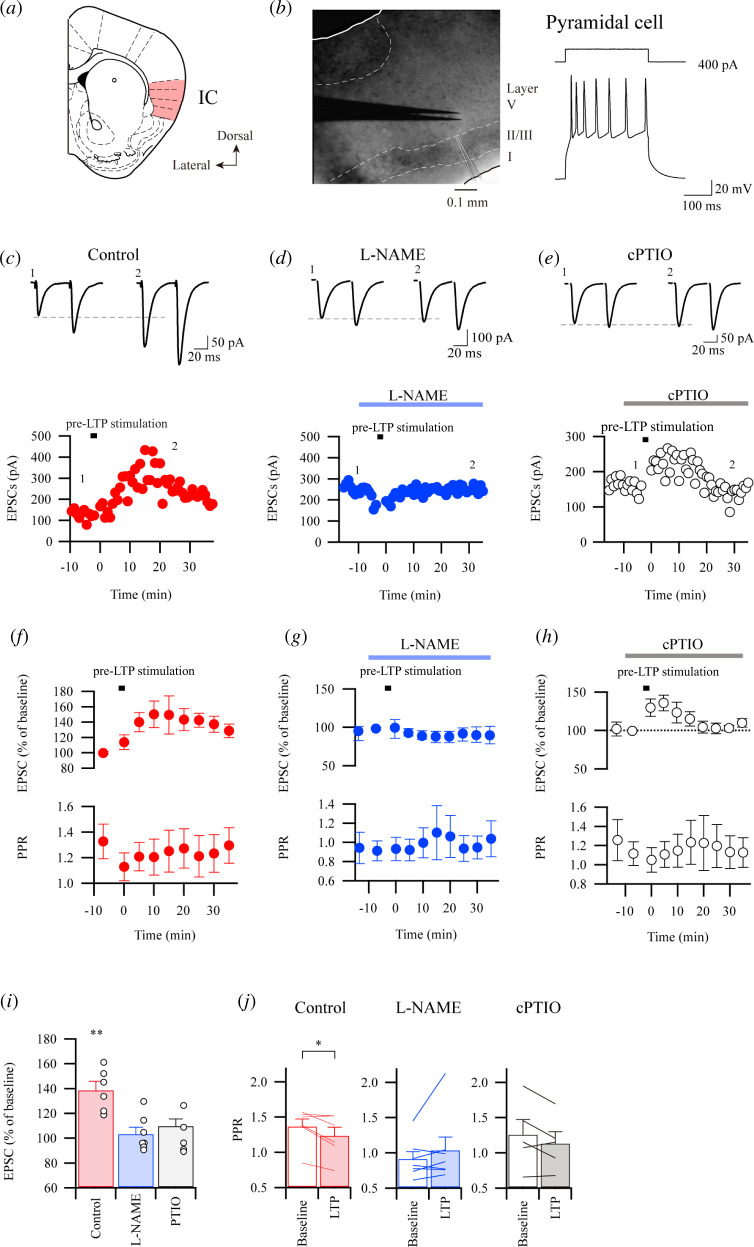
Nitric oxide-mediated induction of pre-LTP in IC. (*a*) A sample image containing IC in the coronal section. (*b*) A microscopic image obtained by differential interference contrast infrared video microscopy (left) and a representative firing (right, lower) induced by depolarizing current pulse injection (300 ms) to the pyramidal cell (right, upper). (*c–e*) Example traces and time course plots of first EPSCs with paired-pulse stimulation without drug application (red closed circle, (*c*) and during application of L-NAME (100 µM, blue closed circle, (*d*) and cPITO (300 µM black open circle, (*e*) before and after the stimulus protocol for induction of pre-LTP. eEPSC with paired-pulse stimulation at 50 ms inter-stimulus interval during baseline (1) and approximately 30 min after the pre-LTP (2) at the holding membrane potential of −75 mV. (*f–h*) Time course of averaged eEPSC amplitudes (upper) and paired pulse ratios (PPRs; lower) of control (*f*) and the effect of L-NAME (*g*) and cPTIO (*h*) application on LTP induction. Note that PPRs in the control decreased after the pre-LTP stimulus protocol. (*i*) Summary of normalized EPSCs in control, L-NAME and cPTIO (control, *n* = 6, *t*_5_ = 5.921, **p = 0.002; L-NAME, *n* = 7, *t*_6_ = 0.1241, *p* = 0.2484; cPTIO, *n* = 5, *t*_4_ = 0.1.781, *p* = 0.1496, paired *t*‐test). (*j*) PPRs before and after pre-LTP stimulation in control (left), L-NAME (centre) and cPTIO (right, control, *n* = 6, *t*_7_ = 2.825, *p = 0.0369; L-NAME, *n* = 7, *t*_6_ = 0.441, *p* = 0.6746; cPTIO, *n* = 5, *t*_4_ = 1.719, *p* = 0.1608, paired *t*‐test). The error bar represents s.e.m.

In previous studies, we have demonstrated that the excitatory synapses in the IC showed post-LTP [15,19]. Here, we like to focus on pre-LTP in the IC. First, we examined whether the excitatory synaptic response can be induced by stimulating layer V in the IC. Identification of the localization of excitatory sources in layer V projecting to layers II/III pyramidal cells was found (figure 1; see §2). Excitatory synaptic responses evoked by the electrodes placed in layer V were observed in layer II/III pyramidal cells which showed typical firing.

First, we investigated pre-LTP in the IC. After achieving a stable baseline recording in response to paired-pulse stimulation (inter-pulse interval of 50 ms) for at least 10 min, we applied the stimulus protocol for pre-LTP (2 Hz for 2 min) at a holding potential of −75 mV [6]. We found that synaptic responses were significantly potentiated, and such potentiation persisted for at least 30–35 min (138.9 ± 6.9% of baseline; figure 1*c,f*). The PPR time course demonstrated that PPR changed during LTP (figure 1*f* lower). Second, we would like to know if pre-LTP in the IC may require NO. To clarify this possibility, we applied an NOS inhibitor, L-NAME (100 µM). As shown in figure 1*d,g*, interestingly, pre-LTP was completely blocked by the L-NAME application (95.8 ± 11.9% of baseline; figure 1*g*). Third, we applied an NO scavenger, cPTIO (300 µM), to block the NO pathway. There was little observation of the elevation of eEPSC amplitude during L-NAME after the pre-LTP protocol. Unlike the application of an NOS inhibitor, the increment of amplitude was induced initially after the stimulus protocol; however, the enhancement was abolished by applying cPTIO (110.1 ± 5.5% of baseline) approximately 20 min after the stimulation (figure 1*h*). Here, figure 1*i* shows the summarized results of normalized amplitude after 30 min of pre-LTP stimulation. There were significant differences in EPSCs between baseline and the period of 30–35 min after the stimulus protocol in the control. There were few differences in eEPSCs that applied L-NAME and cPTIO. These results demonstrated that the NO is necessary for pre-LTP induction in the IC. Significant changes in PPRs before and after LTP stimulation were shown in the control, but not L-NAME and cPTIO, suggesting that the LTP induced by pre-LTP stimulus protocol is mediated by pre-synaptic mechanisms (figure 1*j*).

**Figure 2.**
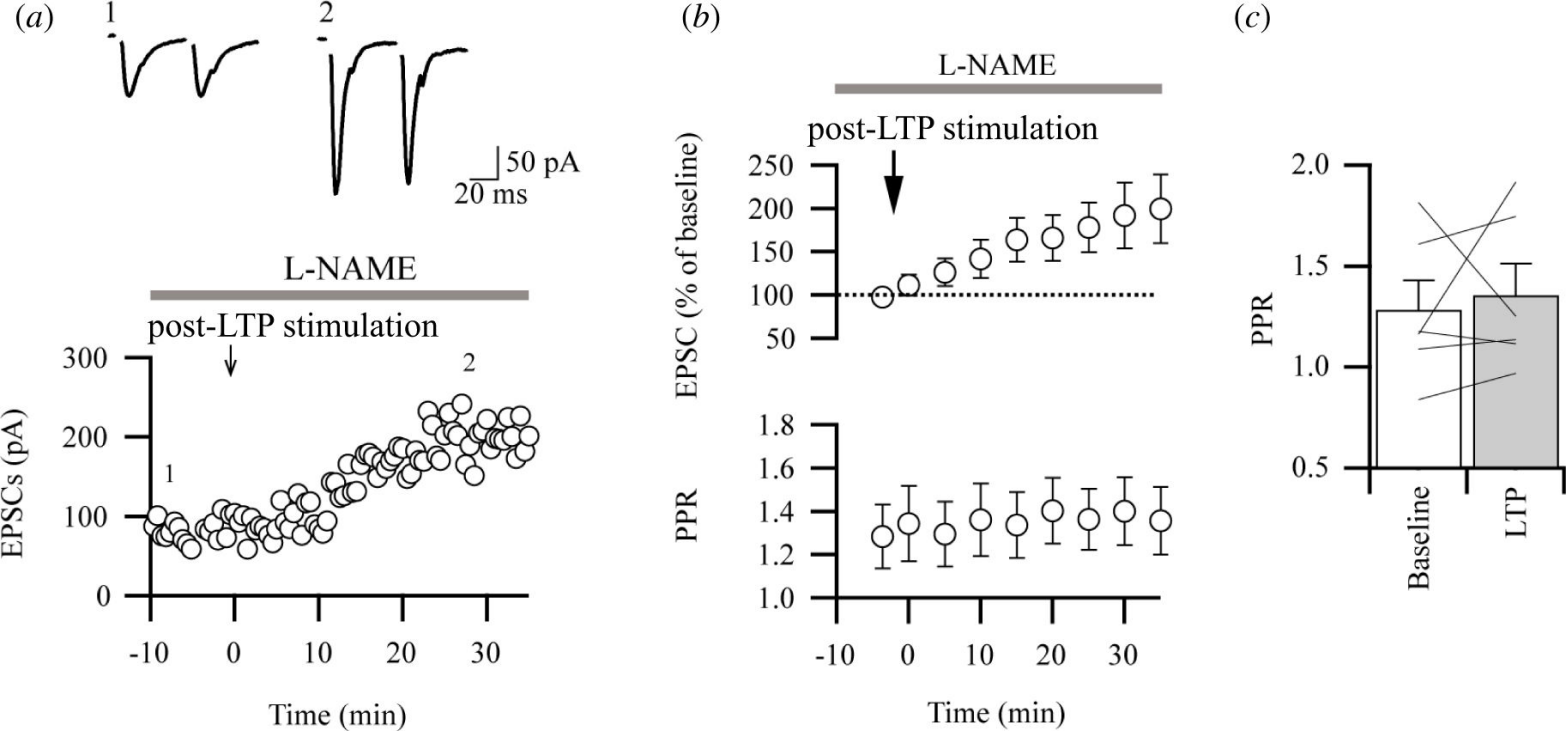
Little effect of NOS inhibitor on post-LTP induction. (*a*) Sample waveforms and time course plots of eEPSC amplitude with paired-pulse stimulation at 50 ms inter-stimulus interval in the baseline and after the stimulus protocol for induction post-LTP. L-NAME (100 µM) started to be applied before recording. (*b*) Time course plots of averaged plots of EPSCs (upper) and PPRs (lower) during baseline and after the stimulus protocol (EPSCs, *n* = 6, *t*_5_ = 2.591, *p = 0.049, paired *t*‐test). Note that the changes in EPSC kinetics were observed after the stimulus protocol for LTP induction. (*c*) Summary data of PPR in post-LTP (Post-LTP, *n* = 6, *t*_5_ = 0.431, *p* = 0.685, paired *t*‐test).

To examine whether NO is also required for post-LTP in the IC, we tested the effects of L-NAME on post-LTP stimulus protocol induced by the pairing protocol [6]. We found that L-NAME has no effect on post-LTP (figure 2*a*). Although the remarkable elevation of eEPSC was observed 30–35 min after post-LTP stimulus protocol, there were no significant changes in PPRs after post-LTP induction in the IC (figure 2*b,c*). These data suggest that, unlike NO-mediated pre-LTP induction, the LTP induced by post-LTP stimulus protocol is mediated by postsynaptic mechanisms and is little affected by the NOS inhibitor.

### (b) Induction of presynaptic long-term potentiation depends on kainate receptors

**Figure 3.**
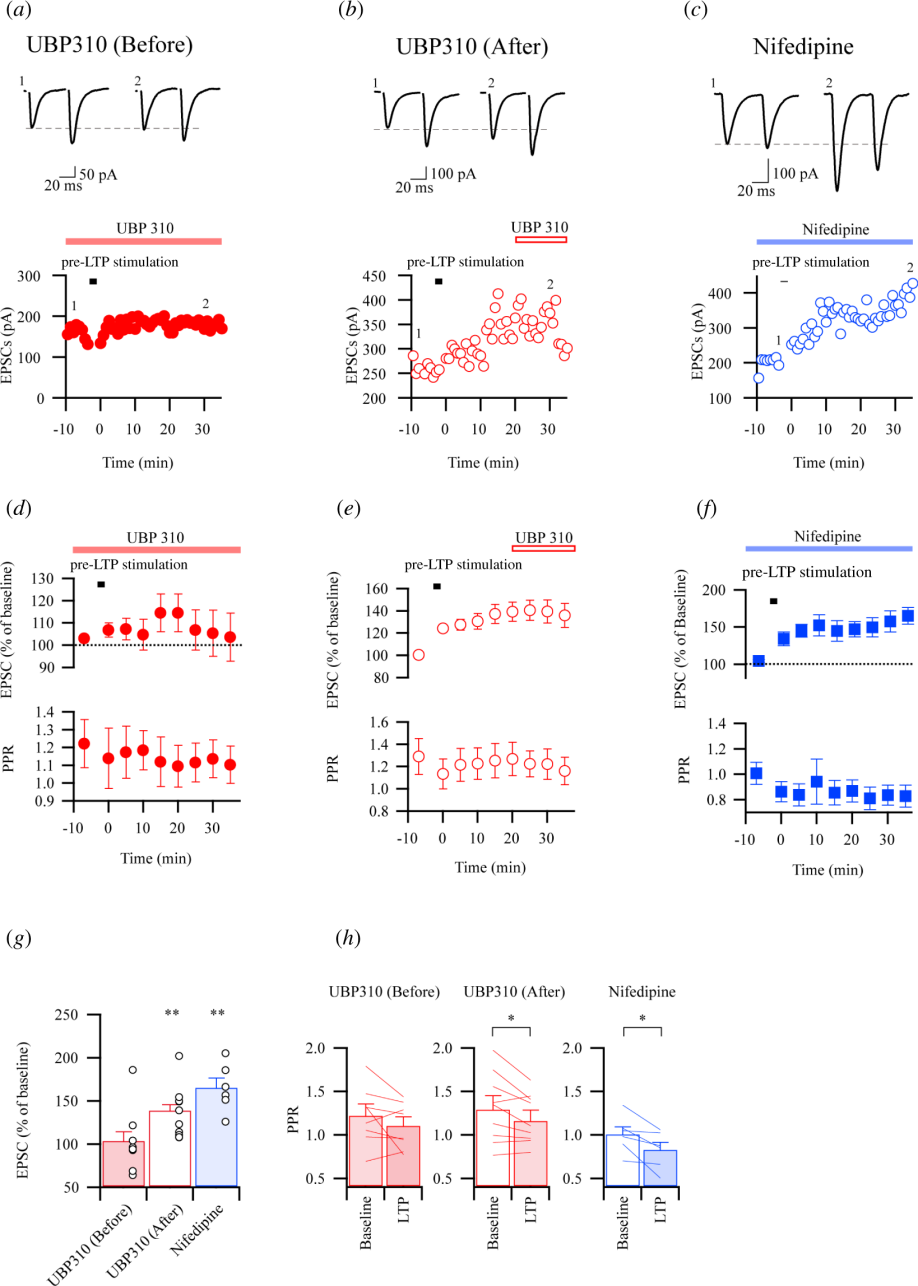
Pharmacological analysis for induction and maintenance of pre-LTP. (*a–c*) Example traces and time course plots of EPSCs during application of UBP310 (10 µM) added to ACSF before (UBP310 before, red closed circle, (*a*) or after (UBP310 after, red opened circle, (*b*) the stimulus protocol for induction of pre-LTP, and application of nifedipine (20 µM, nifedipine, blue circle, (*c*) before the stimulus protocol. (*d–f*) Time course of eEPSC amplitudes (upper) and paired pulse ratios (PPRs; lower) of the effects of UBP310 application before (*f*) and after (*e*), and nifedipine application (*f*) on LTP induction. Summary of normalized EPSCs in UBP310 before, UBP310 after and nifedipine (UBP310 before, *n* = 8, *t*_7_ = 0.03695, *p* = 0.9716; UBP310 after, *n* = 10, *t*_6_ = 3.855, **p = 0.0039; nifedipine, *n* = 6, *t*_5_ = 5.522, **p = 0.0027, paired *t*‐test). (*h*) PPRs of UBP310 before, UBP310 after and nifedipine before and after the protocols for LTP induction (UBP310 before, *n* = 8, *t*_7_ = 1.428, *p* = 0.1963; UBP310 after, *n* = 10, *t*_9_ = 2.771, **p = 0.00217; nifedipine, *n* = 6, *t*_5_ = 2.991, **p = 0.00304, paired *t*‐test).

Calcium entry through the opened receptors indirectly activates NOS [9]. A previous report focusing on pre-LTP also suggests KARs as another candidate for enabling the permeation of extracellular Ca^2+^ into the membrane [5]. In addition, it was shown that the L-type voltage-dependent calcium channel (VDCC) is likely to play an essential role in forming NMDAR-independent LTP in hippocampal synapses [21]. In the anterior cingulate cortex (ACC), Koga *et al*. reported that L-VDCCs also contribute to pre-LTP [6]. Thus, we would like to test the possibility that pre-LTP in the IC can be blocked by the application of KAR and L-type VDCC antagonists. Figure 3*a,b* show the effect of the KAR antagonist before and after the stimulus protocol. UBP310 (10 µM) was added before the LTP-induced stimulus protocol blocked the induction of pre-LTP, but it had little effect on the maintenance of pre-LTP when the drug was added after the induction (figure 3*d,e,h*). Interestingly, the L-type VDCC blocker nifedipine (20 µM), which was applied before the application of the LTP-induction protocol, had little effect on pre-LTP (figure 3*c,f,g*). The results indicate that KARs play a selective role in the induction of pre-LTP, but L-type VDCCs are not involved in pre-LTP.

### (c) Increasing nitric oxide production enhances presynaptic glutamate release

**Figure 4.**
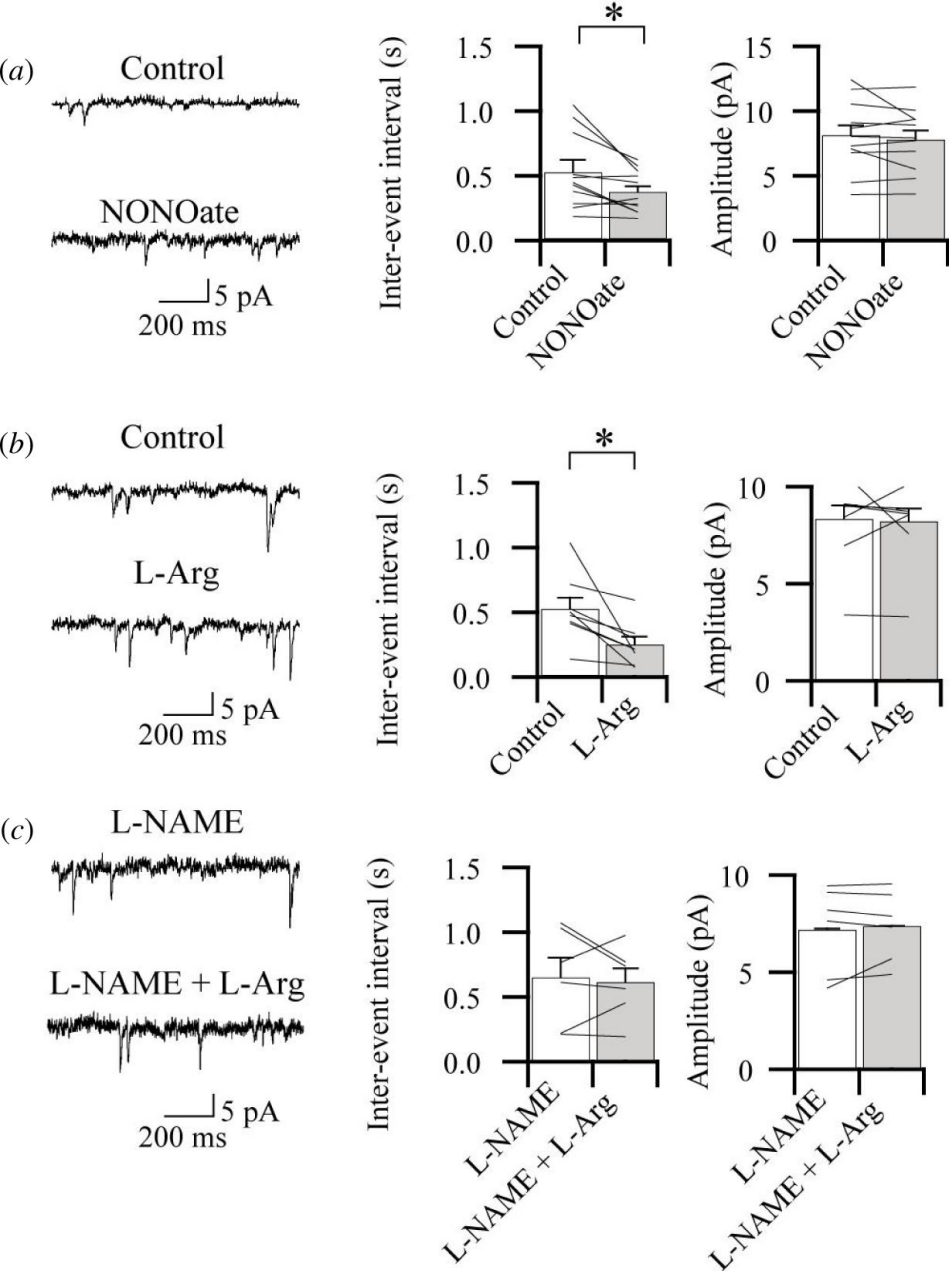
NO modulates release machinery in excitatory synapses and is provided by the presynaptic site. (*a*) and (*b*) Effects of NO donner and substrate for NO synthase on miniature EPSC (mEPSC). Sample traces showing mEPSC responses before (Control) and during application of spermine NONOate (NONOate, 100 µM left in *(a*) and L-arginine (L-Arg, 1 mM left in (*b*). Averaged inter-event intervals (IEI) and amplitude of mEPSC during application of NONOate (middle and right in (*a*) and L-Arg (middle and right in (*b*). There were significant differences in mEPSC events when comparing the control and after the application of NONOate (*n* = 12, t_(11)_ = 2.381, *p = 0.036, paired *t*‐test) and L-Arg (*n* = 8, t_(7)_ = 2.667, *p = 0.032, paired *t*‐test). There were little significant differences in mEPSC amplitude when comparing the control and after the application of NONOate (t_(11)_ = 1.165, *p* = 0.268) and L-Arg (t_(11)_ = 0.246, *p* = 0.813, paired *t*‐test). (*c*) Suppressive effects of NOS inhibitor on L-Arg-induced enhancement of mEPSC events. Sample traces showing mEPSC responses during NOS inhibitor, L-NAME (100 µM), and additional application of L-Arg (1 mM left in (*c*)). Averaged events and amplitude of mEPSC during L-NAME (middle) and additional administration of L-Arg (right). There were no differences in frequency and amplitude of mEPSC (frequency, *n* =5, t_(5)_ = 0.504, *p* = 0.636; amplitude, t_(5)_ = 0.698, *p* = 0.516, paired *t*‐test).

It has been reported that NO enhanced mEPSCs in hippocampal neurons [22]. Since NO is essential for the induction of pre-LTP, we wanted to know if NO precursors may affect the frequency of mEPSCs, a measurement of presynaptic glutamate release [23]. In the bath application of NO donner spermine NONOate (NONOate; 100–200 µM), the effect is selective on frequency and the amplitudes were not affected (figure 4*a*–*c*). We also repeated experiments with L-Arg, a substrate for NO synthase. Similarly, L-Arg also produced the same results. Furthermore, the effect of L-Arg was completely blocked by a NOS inhibitor L-NAME (100 µM). These data indicate that NO released by NO donner and L-Arg application affects presynaptic release machinery but does not affect the postsynaptic membrane figure 4.

### (d) Nitric oxide is required for presynaptic long-term potentiation between a pair of pyramidal cells in the insular cortex

**Figure 5.**
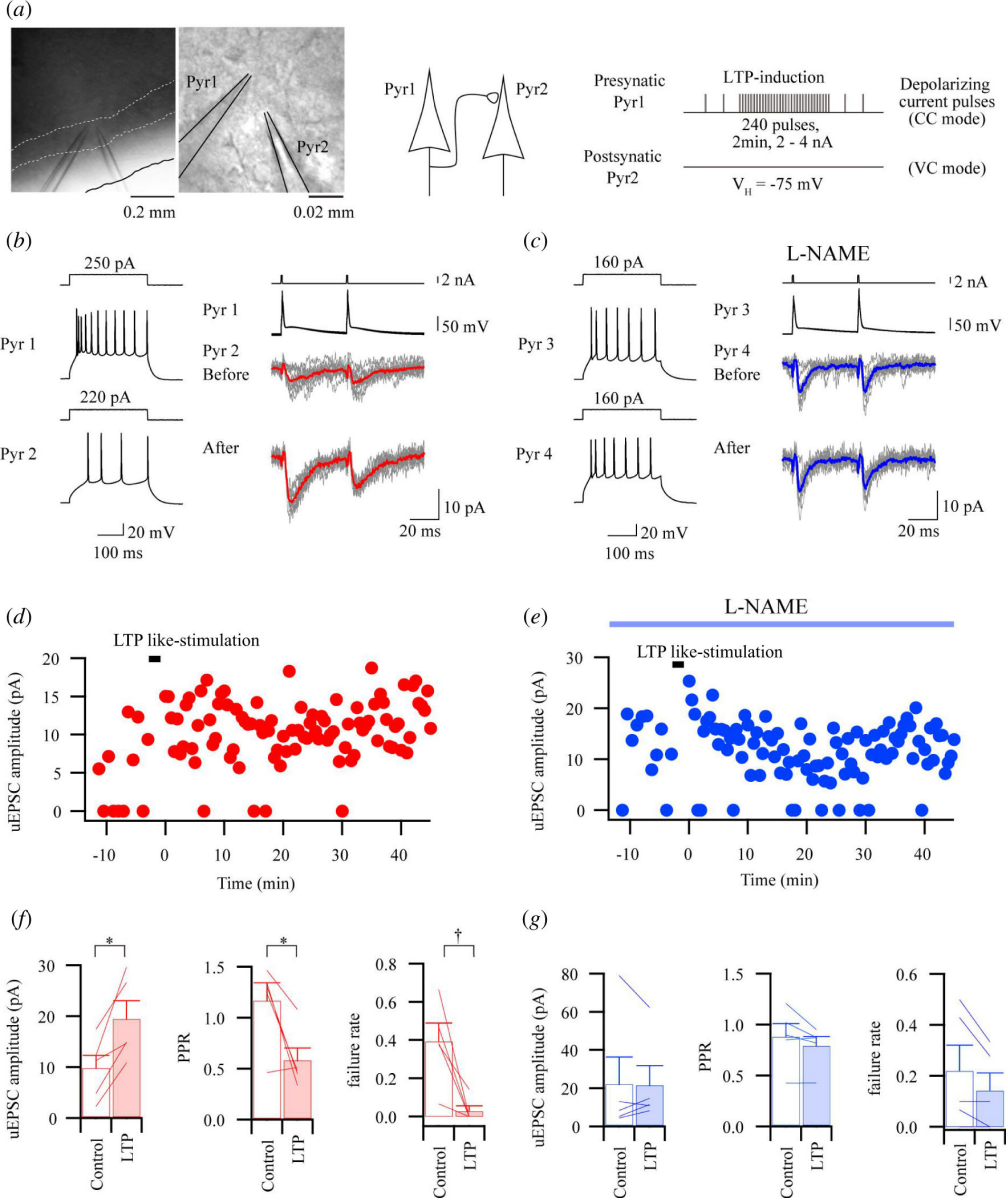
Pre-LTP in unitary EPSC (uEPSC) responses that were obtained from the excitatory connection between pyramidal cells. (*a*) Double whole-cell patch-clamp recordings were performed in layer II/III of IC under differential interference contrast infrared video microscopy (DIC, left). Experimental protocol of uEPSC events for induction of pre-LTP (right). (*b*) Pre-LTP-like responses appeared in the excitatory synaptic connection between Pyr1 and Pyr2. Firing properties of Pyr1 and Pyr2 were induced by depolarizing current pulse injections (300 ms) to the neurons shown in (*a*, left). Postsynaptic uEPSCs were recorded from Pyr2 before (the second trace from the bottom) and after (bottom trace) the stimulus protocol for pre-LTP. uEPSC responses were induced by paired depolarizing current pulses to Pyr1 (top traces) to induce action potentials (the second traces from the top). Grey and red waveforms indicate 11 consecutive traces and their averaged traces, respectively. Note that no failure responses were observed in pre-LTP-like responses after the stimulus protocol. (*c*) Pre-LTP-like responses were blocked by the NOS inhibitor. Firing properties of Pyr3 and Pyr4 (left). uEPSCs recorded from Pyr4 before and after the stimulus protocol to induce pre-LTP NOS inhibitor, L-NAME was applied before recording (right). Grey and blue waveforms indicate 11 consecutive traces and their averaged traces, respectively. (*d*) and (*e*) Time course plots of uEPSCs obtained from Pyr2 and Pyr4 showing in (*b*) and (*c*). (*f*) Summary of data showing uEPSC amplitude, PPR and failure rate (uEPSCs, *n* = 5, *t*_4_ = 3.625, *p = 0.022; PPR, *t*_4_ = 3.102, *p = 0.036, paired *t*‐test; failure rate, †*p* = 0.043, Wilcoxon test). (*g*) Summary of data showing uEPSC amplitude, PPR and failure rate with the application of L-NAME (*n* = 5, *t*_4_ = 0.117, *p* = 0.912; PPR, *t*_4_ = 1.809, *p* = 0.145, paired *t*‐test; failure rate, *p* = 0.109, Wilcoxon test).

It is presumed that employed electrical stimulus protocol, even minimal stimulation by an electrode, simultaneously activates various and unidentified fibres and presynaptic neurons projecting to postsynaptic neurons. Consequently, it is hardly possible to identify the origins of presynaptic fibres and neurons. The double-patch clamp technique is one of the best methods to determine the presynaptic neurons and enables us to record cortico-cortical-mediated EPSC directly. Therefore, we try to induce LTP-like responses in the cortico-cortical synaptic transmission in excitatory connections by the double-patch clamp technique. A sample digital image correlation (DIC) image of double patch pipettes shows that the connecting neuron pairs exist within approximately 20 µm (figure 5*a*). In this study, the connection rate in pairs between pyramidal cells was 9.3% (10 connections in 108 trials). LTP-like responses of uEPSC were induced by repetitive action potentials provided by depolarizing pulses to the presynaptic pyramidal cell. The plotted amplitude obtained from the same cell in figure 5*b* demonstrated a decrease in failure responses after the stimulus protocol (figure 5*d*). Figure 5*f* shows the summaries of pooled data and that there are significant differences in uEPSC amplitude, PPR and failure rate. On the other hand, L-NAME applied before the stimulus protocol suppressed the induction of LTP-like responses in uEPSCs (figure 5*c*). In the group that applied L-NAME, there were no significant changes in uEPSCs, PPR or failure rate (figure 5*g*). These results also suggest that LTP-like responses not only have NO-dependency but also are induced in the cortico-cortical synaptic transmission composed by a pair of pre- and post-synaptic IC pyramidal cells.

## 4. Discussion

In this study, we demonstrate a novel form of pre-LTP in the adult IC. Induction of pre-LTP was blocked by not only KAR antagonists but also the NOS inhibitor. Using a double patch-clamp recording technique, we showed that such pre-LTP appeared in cortico-cortical synapses between cortical pyramidal cells. The technique combined with the pharmacological approach also revealed that presynaptic NMDARs contributed to inducting the LTP. While the induction of pre-or post-LTP requires the activation of glutamate NMDAR and KAR as previously reported in the ACC and IC [6,24], our study indicated that the expression of this pre-LTP requires NOS activity. To our knowledge, this is the first reported form of LTP that requires NOS activity for potentiation. Together with recent studies, this work demonstrates that cortical circuits employ different forms of LTP during essential sensory, emotional and memory functions [13,25–30].

Many mechanisms underlying LTP have been proposed in cortical areas. Previous reports and reviews demonstrated that various signal pathways in the pre- and/or post-synaptic sites participate in the induction of LTP. In the postsynaptic site, AMPARs have heteromeric assemblies of different subunits and are triggered by NMDAR activation. The receptors play a critical role in the induction of post-LTP in the ACC and visual cortex [31]. On the other hand, several types of mechanisms for the induction of pre-LTP observed in the hippocampus, amygdala and ACC were also proposed [5,32,33]. In the cortical area, the AC1-PKA pathway activated by KARs in presynaptic excitatory sites contributes to the elevation of glutamate release [6]. In our study, the application of UBP310 before the stimulus protocol for LTP completely suppressed the induction of LTP but the UBP310 after the stimulus protocol failed. These results suggest the possibility that the recruitment of KARs is necessary to induce pre-LTP in the IC. It is known that KARs stimulate the AC-PKA pathway and play an essential role in the induction of pre-LTP in the ACC [2,5,6]. Two signalling pathways of KAR have been proposed: canonical ionotropic and non-canonical metabotropic pathways [34]. Previous results indicated that the KAR-mediated current was slight but characterized by an outward rectifier [35]. In addition, pre-LTP in ACC and hippocampus depends on L-type VDCC [36,37]. Our results exhibited that pre-LTP has little sensitivity to the L-type VDCC blockers, indicating that two channels are excluded from the candidates of induction. Thus, the mechanism of the presynaptic KAR-NO pathways does not resemble those previously found and reported in cortical regions and other brain areas.

NO-mediated LTP has already been observed in excitatory synapses in many brain regions. Although it requires elevation of [Ca^2+^]_i_ as a trigger of NO synthesis, several pathways of NOS activation and consequent LTP-induction were hypothesized. Our previous immunobiological analysis exhibited neuronal NOS is abundantly placed in the IC [38]. It is also reported that activation of neuronal NOS is catalysed by at least three protein kinases, the phosphatidylinositol 3-kinase/protein kinase B (Akt/PKB), Ca^2+^/calmodulin-dependent protein kinase II (CaMKII) or protein kinase A (PKA) [39]. Therefore, it is presumed that induction of the pre-LTP requires Ca^2+^ influx through KAR and phosphorylation of nNOS brought about by AC-PKA through KAR. Thus, future studies should focus on understanding the downstream pathway of KAR for pre-LTP.

In addition, NO scavenger, cPTIO, blocked the induction of pre-LTP, indicating that the induction of LTP may be affected by NO. The frequency of mEPSC was also increased by the application of NONOate and L-Arg (figure 4). These results also support the hypothesis that NO plays an essential role in modulating transmitter release in local IC excitatory synapses. The differences between the mechanism of pre-LTP in the IC and other cortical areas suggest that cortical synapses are not using the same mechanisms for LTP.

Because *in vivo* two-photon microimaging showed that only approximately 20% of IC neurons in response to both painful and anxious-related stimulations are overlapped, pre- or post-LTP in the IC may separately develop in different neurons [40]. Anxiety disorder is common in chronic pain patients [41]. Experimental studies and clinical research also showed a strong relationship between chronic pain and anxiety disorder [6,42]. Our results strongly suggest that cortical areas which are critical for pain perception and modulation are highly plastic, and future understanding of basic mechanisms for these different forms of plasticity will provide new clinical strategies to relieve chronic pain and its related emotional disorders.

## Data Availability

Data are provided as the electronic supplementary material [43].
